# Supplementary material for: Lentinula edodes Cultured Extract and Rouxiella badensis subsp. acadiensis (Canan SV-53) Intake Alleviates Immune Deregulation and Inflammation by Modulating Signaling Pathways and Epigenetic Mechanisms
Source: Int J Mol Sci. 2023 Sep 27;24(19):14610. doi: 10.3390/ijms241914610 (PMC10572597; doi:10.3390/ijms241914610)
Supplement: Supplementary file 1 [file ijms-24-14610-s001.zip › ijms-2596757-supplementary.pdf]

Effect of Treatment on DNA Methylation Status in Small Intestine of Mice

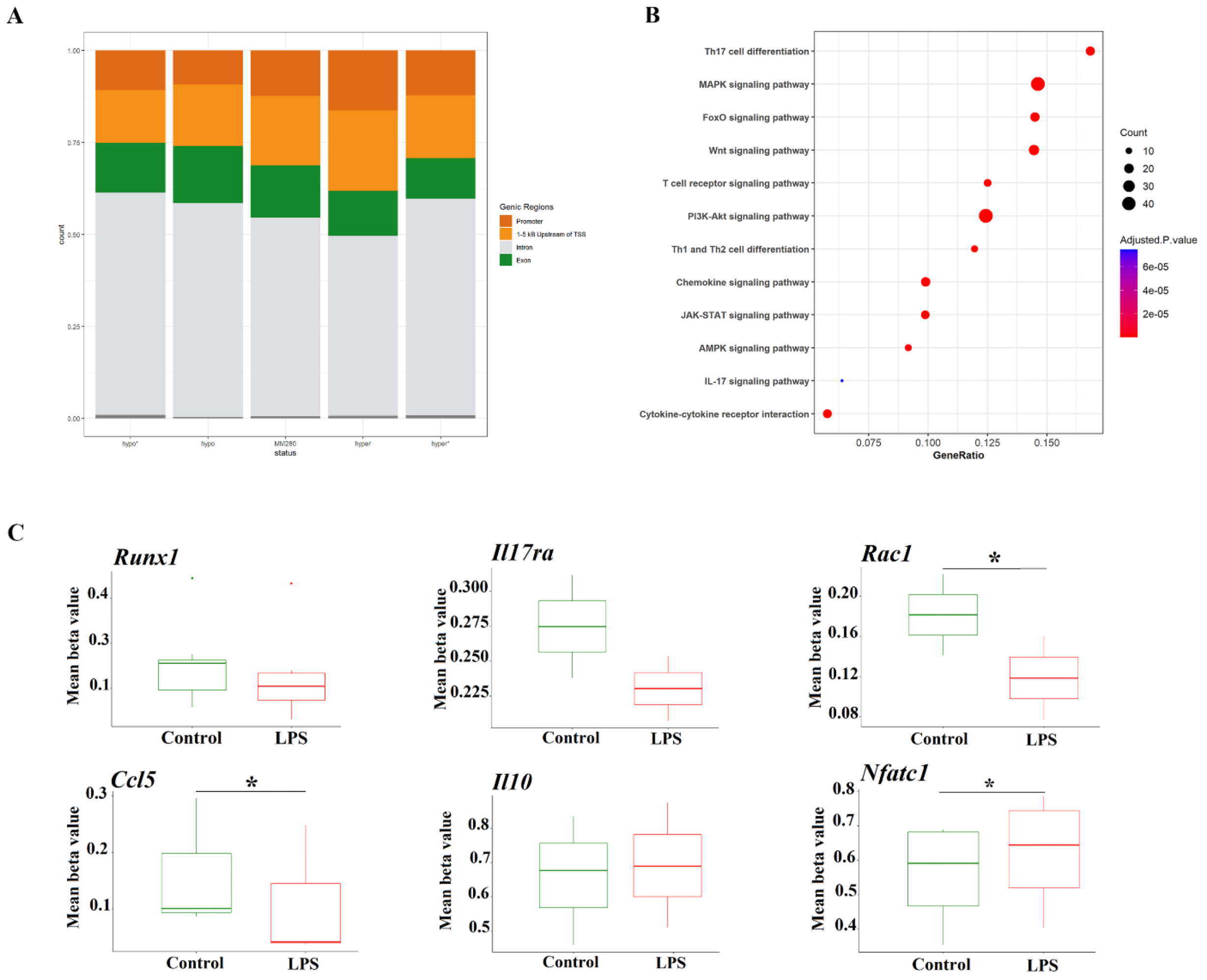

Figure S1. DNA methylation analysis in adult mice four weeks after LPS injection at puberty. Mice received AHCC (2gr/kg BW/d) in drinking water or drinking water without AHCC for two weeks, one week before and one week after LPS injection. (**A**) Genomic regulatory regions, where the hypo and hyper columns indicate all hypo- and hypermethylated DMRs, respectively and hypo* and hyper* columns represent significant hypomethylated and hypermethylated DMRs, respectively. MM280 column represents the overall distribution of the array. (**B**) Pathways enrichment analysis visualized by Enrichr, (**C**) Boxplots of differentially methylated genes.*p<0.05 vs. control.
